# Prediction of Population Size and Ageing in China Based on the Leslie Matrix Model

**DOI:** 10.1093/geroni/igaf122.2303

**Published:** 2025-12-31

**Authors:** Juan Luo, Keting Xia, Chiyue Huang

**Affiliations:** Shanghai University of Engineering Science, Shanghai, Shanghai, China (People’s Republic); Shanghai University of Engineering Science, Shanghai, Shanghai, China (People’s Republic); Shanghai University of Engineering Science, Shanghai, Shanghai, China (People’s Republic)

## Abstract

Objective To better understand the changes in population size and ageing trends in China and to provide theoretical and policy support for China’s future response to the various impacts triggered by heavy population ageing, we predict China’s future population and the degree of ageing. Methods The age-specific fertility and mortality data of the China Population and Employment Statistical Yearbook from 2006 to 2023 were collated, three fertility scenarios—low, medium, and high—were established, and their future values were predicted by using EXCEL; the age-specific population data published by the National Bureau of Statistics (NBS) for the year 2022 were used to predict the future total population of China by using the Leslie Matrix Model in conjunction with the migration rate. Total population to make predictions. Conclusion Under the three scenarios, the total population of China showed a downward trend. Under the middle plan, the total population will fall below 1.4 billion in 2036 and 1.3 billion in 2049. The decline trend changes from a gentle decline in 2023-2039 to a steep decline around 2040, showing a rapid downward trend. The total size of China’s population will continue to decline in the next 30 years; The degree of ageing continues to deepen, and will exceed 21 per cent in 2032, entering a severely ageing society.

